# Soluble egg antigens of *Schistosoma japonicum* induce senescence in activated hepatic stellate cells by activation of the STAT3/p53/p21 pathway

**DOI:** 10.1038/srep30957

**Published:** 2016-08-04

**Authors:** Jinling Chen, Jing Pan, Jianxin Wang, Ke Song, Dandan Zhu, Caiqun Huang, Yinong Duan

**Affiliations:** 1 Department of Pathogen Biology, School of Medicine, Nantong University, Nantong 226001, Jiangsu, People’s Republic of China; 2 Laboratory Medicine Center, Affiliated Hospital of Nantong University, Nantong 226001, Jiangsu, People’s Republic of China; 3 Orthopedics and Traumatology Center of PLA, The 153rd Central Hospital of People's Liberation Army, Zhengzhou 450042, Henan, People’s Republic of China

## Abstract

Liver fibrosis is characterized by the activation of hepatic stellate cells (HSCs). Recent findings suggest that senescence of activated HSCs might limit the development of liver fibrosis. Based on previously observed anti-fibrotic effects of soluble egg antigens from *Schistosoma japonicum in vitro,* we hypothesized that SEA might play a crucial role in alleviating liver fibrosis through promoting senescence of activated HSCs. We show here that SEA inhibited expression of α-SMA and pro-collagen I and promoted senescence of activated HSCs *in vitro*. In addition, SEA induced an increased expression of P-p53 and p21. Knockdown of p53 inhibited the expression of p21 and failed to induce senescence of activated-HSCs. Phosphorylated STAT3 was elevated upon SEA stimulation, while loss of STAT3 decreased the level of p53 and senescence of HSCs. Results from immunoprecipitation analysis demonstrated that SOCS3 might be involved in the SEA-induced senescence in HSCs through its interaction with p53. This study demonstrates the potential capacity of SEA in restricting liver fibrosis through promoting senescence in HSCs. Furthermore, a novel STAT3-p53-p21 pathway might participate in the observed SEA-mediated senescence of HSCs. Our results suggest that SEA might carry potential therapeutic effects of restraining liver fibrosis through promoting senescence.

Liver fibrosis, an outcome of chronic liver injury, is characterized by an activation of hepatic stellate cells (HSCs) and excessive accumulation of extracellular matrix (ECM) proteins. The activated HSCs express α-smooth muscle actin (α-SMA) and produce large amount of collagen, which is the central pathogenic event of liver fibrosis^1^,^2^. The accumulation of ECM proteins and the aggregation of collagen could gradually lead to liver damage through the formation of fibrous scars, which could eventually develop into cirrhosis^3^. Once established, fibrosis is considered to be irreversible. Nevertheless, many reports have recently demonstrated that liver fibrosis of various etiologies could be improved to a certain degree if the pathogenic agents were obliterated^4^,^5^. In case of liver fibrosis, the impactful tactic for resolving the disease would be to inhibit the activation of HSCs and reduce the secretion of ECM. In addition, accelerating the clearance of activated HSCs could also be of vital significance^6^,^7^.

Based on recent findings, senescence of activated HSCs might play a role in limiting the development of liver fibrosis by markedly inhibiting the proliferation of HSCs and significantly reducing the expression of fibrogenic proteins^8^. Some important features of senescence include irreversible growth arrest, enlarged cellular morphology and expression of senescence-associated beta-galactosidase (SA-β-Gal). Cells are induced to undergo senescence mainly through activation of the p53 and the p16/Rb pathways. In general, p53 promotes senescence through activation of cyclin-dependent kinase inhibitors, such as p21; whereas p16^INK4α^ inhibits cyclin-dependent kinases^9^. Furthermore, it has been well established that some cytokines, such as IL-6, IL-8, IL-10 and IL-22, as well as their downstream signaling molecules, including signal transducer and activator of transcription (STAT) and suppressor of cytokine signaling (SOCS), could promote senescence in many different cell types^10^.

Although the leading causes of liver fibrosis include chronic HCV infection, alcohol abuse, and nonalcoholic steatohepatitis (NASH), infection of *Schistosoma japonicum (S. japonicum*) has been shown to be an important etiological factor of liver fibrosis in Asian countries^11^,^12^. Previous studies have demonstrated that the eggs of *Schistosoma mansoni* and *S. japonicum* could induce a quiescent morphology and anti-fibrogenic phenotype in LX-2 cells, which could ultimately block fibrogenesis^13^,^14^. Importantly, soluble egg antigens (SEA) from *Schistosoma* might be capable of promoting immunocyte apoptosis^15^ and therefore, could play a crucial role in alleviating liver fibrosis through inducing quiescent morphology in HSCs^14^. In accordance with these aforementioned studies, our previous reports have also demonstrated that SEA from *S. japonicum* limits liver fibrosis through inhibiting the activation and promoting the apoptosis of LX-2 cells^1^,^16^. In this study, we further investigated the role of SEA in restraining liver fibrosis through promoting senescence of activated HSCs.

## Results

### SEA inhibits the expression of liver fibrosis-associated proteins

**Figure 1:**
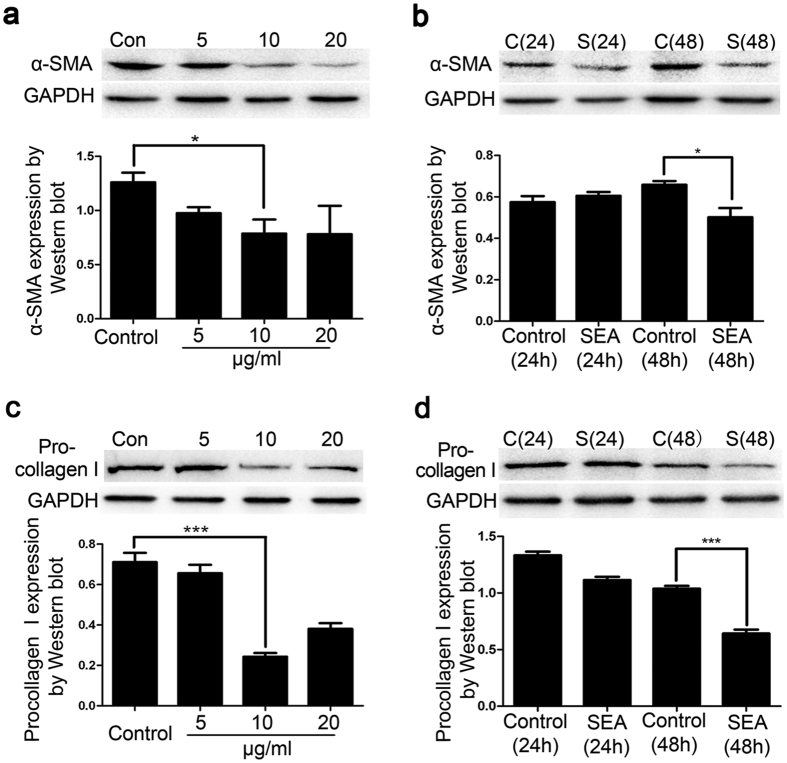
SEA inhibits the expression of liver fibrosis-associated proteins. (**a,c**) Western blots showing the expression of α-SMA and pro-collagen I after treatment with several doses of SEA. GAPDH level is shown as loading control. (**b,d**) Following SEA (10 μg/ml) treatment for the indicated times, the expression of α-SMA and pro-collagen I was measured by Western blots. Values are mean ± SD/SEM of triplicate experiments. Statistical differences between groups are shown as follows: *p < 0.05; ***p < 0.001.

To determine the effects of SEA on liver fibrosis *in vitro*, we examined the protein expression of α-SMA and procollagen I, which are widely accepted as fibrosis markers in activated HSCs^17^. As shown in Fig. 1a,c, LX-2 cells were exposed to several doses of SEA at various concentrations (0, 5, 10 and 20 μg/ml). Expression of α-SMA and pro-collagen I in LX-2 cells was noticeably reduced after treatment with SEA at 10 μg/ml. We also measured the levels of α-SMA and procollagen I in LX-2 cells treated with SEA for 24 h and 48 h and found that their protein expression was obviously declined after 48 h (Fig. 1b,d). Based on these observations, the SEA treatment at a concentration of 10 μg/ml for 48 h was selected for all the subsequent experiments. These results suggested that SEA might be capable of restricting liver fibrosis through inhibiting the expression of α-SMA and procollagen I.

### SEA inhibits fibrosis by promoting senescence in HSCs

**Figure 2:**
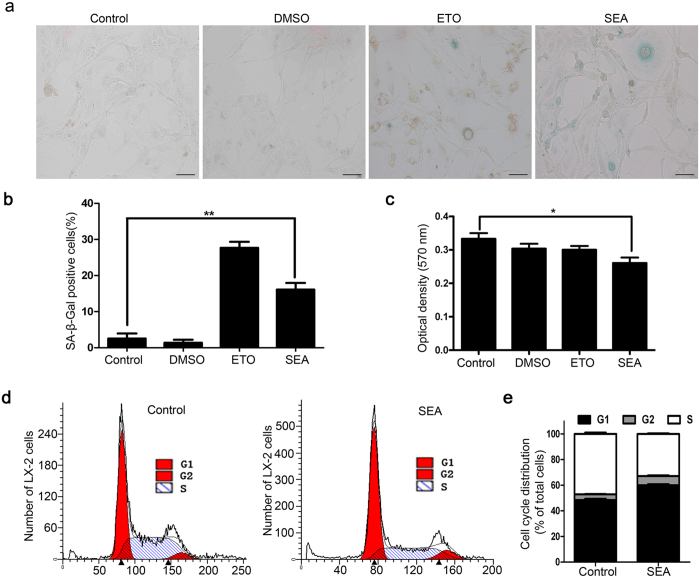
SEA inhibits fibrosis by promoting HSCs senescence. (**a**) SEA-induced senescence as indicated by SA-β-Gal activities. Dimethyl Sulphoxide (DMSO) and Etoposide (ETO) served as the negative and positive control for senescence, respectively. (**b**) Statistical analysis for the percentage of SA-β-Gal positive cells among LX-2 cells treated with or without SEA (10 μg/ml). (**c**) SEA inhibited LX-2 cell proliferation as determined by MTT. (**d,e**) Cell cycle distribution was measured by flow cytometry analysis at 2 days following SEA treatment. Statistical differences between groups are shown as follows: *p < 0.05; **p < 0.01.

Senescence in activated HSCs can contribute to amelioration of liver fibrogenesis^2^,^8^. Therefore, we ask if SEA can exert potential effects on senescence in HSCs. To monitor senescence, SA-β-gal, a senescence-associated marker, was used to examine senescence in LX-2 cells after SEA treatment. As anticipated, we observed high numbers of SA-β-Gal positive cells following 48 h of incubation with SEA (Fig. 2a,b). SEA exposure slightly reduced the proliferation of LX-2 cells as measured by MTT analysis (Fig. 2c). Apart from these observations, more cells were arrested in the G0/G1 phase, accompanied by a decrease in proportion of cells in the S or G2/M phase in the SEA-treated group (Fig. 2d,e). Collectively, these data demonstrated that SEA could promote senescence in activated HSCs, thereby, potentially slowing down liver fibrogenesis.

### Cell senescence occurs at the advanced stage of *S. japonicum* infection

**Figure 3:**
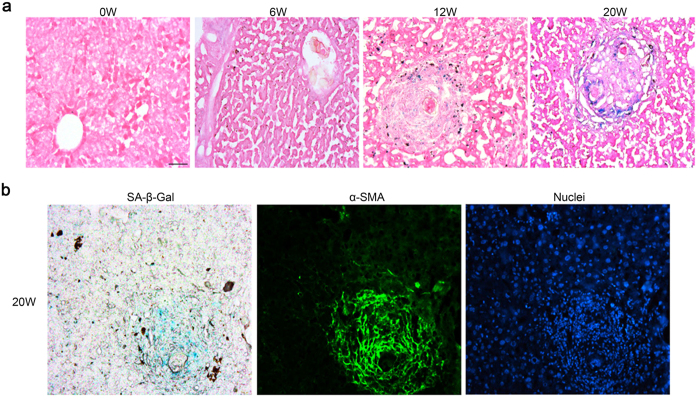
Cell senescence occurs at the advanced stage of *S. japonicum* infection. (**a**) Counterstaining of SA-β-Gal staining (blue) and hematoxylin-eosin staining (red) of liver infected with *S. japonicum*. (**b**) Counterstaining of SA-β-Gal staining (blue) and immunofluorescence of α-SMA (green). Bar: 50 micrometers.

Our previous reports suggest that the deposition of fibrosis-associated proteins, such as α-SMA and collagen, decreases remarkably in liver at the advanced stage of *S. japonicum* infection. Others have observed that as infection progresses, the size of egg granulomas of *S. japonicum* at the advanced phase decreases compared to that at the chronic phase^18^. Furthermore, our present data showed that the number of senescent cells gradually increased with time in mice infected with *S. japonicum* (Fig. 3a). The marker for activated HSCs, α-SMA, was expressed in the peripheral regions of egg granulomas post-*S. japonicum* infection^18^. In addition, SA-β-Gal counterstaining and immunofluorescence both indicated that the senescent cells co-localized with the α-SMA positive cells (Fig. 3b). These data suggested that activated HSCs adopted a senescent phenotype at the advanced stage of *S. japonicum* infection, which could lead to alleviation of liver fibrosis.

### SEA-mediated LX-2 senescence is dependent on the p53 but not the p16 pathway

**Figure 4:**
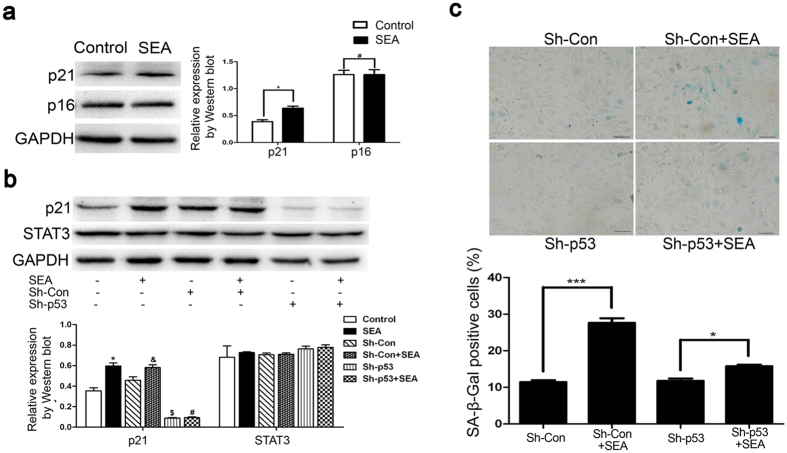
SEA-induced LX-2 senescence is dependent on the p53–p21 axis. (**a**) Expression of p21 and p16 was measured by Western blots. *p < 0.05 compared to the control group; ^#^*p* > 0.05 compared to the control group. (**b**) The expression of p21 and STAT3 was detected by Western blots after LX-2 cells were transfected with p53-targeting Sh-RNA or scrambled Sh-RNA and treated with or without SEA (10 μg/ml). *p < 0.05 compared to the control group; ^&^*p* < 0.05 compared to the Sh-Con group. ^$^*p* < 0.05 compared to the control group; ^#^*p* > 0.05 compared to the Sh-p53 group. (**c**) Knockdown of p53 suppressed SEA-induced senescence as indicated by SA-β-Gal activities. Bar: 50 micrometers. Statistical differences between groups are shown as follows: ***p < 0.001; *p < 0.05.

Next, we explored the potential mechanism of SEA-induced senescence in activated HSCs. The paradigmatic tumor suppressor protein, p53, has been shown to play a critical role in the induction of senescence^19^. As illustrated in Fig. 4a, SEA exposure increased not only p53 as described in our previous study^1^, but p21. Nevertheless, the expression of p16, which is also considered as a critical mediator in the establishment and maintenance of cellular senescence^20^, showed no significant difference in the presence or absence of SEA.

To further explore the molecular pathways underlying SEA-induced senescence, we used shRNA designed specifically against p53 to test the expression of downstream target proteins as described previously^1^. Our results revealed that knockdown of p53 could markedly downregulate the expression of p21 (Fig. 4b). We further investigated the influence of p53 depletion on changes in senescence-related phenotypes. Importantly, the SA-β-Gal activities induced by SEA were reversed by knockdown of p53 (Fig. 4c). Collectively, these results indicated that the p53–p21 axis was essential for SEA-induced senescence, whereas, the p16 pathway was not contributive to the process.

### SEA induces LX-2 senescence via a STAT3-dependent mechanism

**Figure 5:**
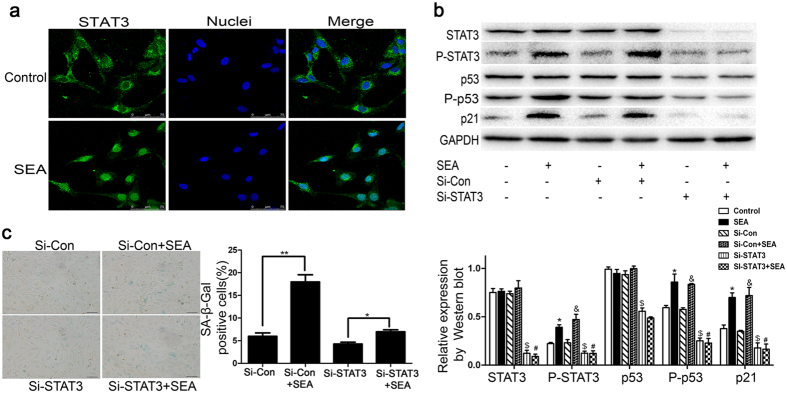
SEA-induced LX-2 senescence is dependent on STAT3. (**a**) The expression of STAT3 in LX-2 cells was determined by immunocytochemistry using a confocal laser scanning microscope, and the nuclei were stained with Hoechst 33342. (**b**) Western blot analysis for STAT3, P-STAT3 (Y705), p53, P-p53 (S15) and p21 proteins in Si-STAT3-treated LX-2 cells. *p < 0.05 compared to the control group; ^&^*p* < 0.05 compared to the Si-Con group; ^$^*p* < 0.05 compared to the control group; ^#^*p* > 0.05 compared to the Si-STAT3 group. (**c**) A representative image from SA-β-Gal staining and percentage (%) of SA-β-Gal -positive cells following Si-Con or Si-STAT3 treatment with or without SEA. Bar: 50 micrometers. Statistical differences between groups are shown as follows: *p < 0.05; **p < 0.01.

Previous studies have demonstrated the controversial role of the STAT3 pathway in liver fibrogenesis. Xu *et al*. suggested that STAT3 signaling led to activation of crosslinking of TGF-β1 in hepatic stellate cells, which could exacerbate liver injury and fibrosis^21^. In contrast, STAT3 activation also appeared to mediate the inhibitory effect of interleukin-22 on mouse liver fibrogenesis^2^. Therefore, we set out to investigate the precise role of the STAT3 pathway on liver fibrosis in human activated HSCs. Western blot analysis demonstrated that phosphorylation of STAT3 at Tyr705, which is a characteristic of the activated form of STAT3 as a transcriptional regulator, increased profoundly in SEA-treated cells, whereas the expression of total STAT3 was not affected. Further, fluorescence staining showed that STAT3 accumulated in the nuclear upon SEA stimulation (Fig. 5a). In agreement with the observed morphological changes, the STAT3 depletion downregulated the expression of cellular senescence-associated proteins in LX-2 cells, including P-p53, p53, and p21 (Fig. 5b). To clarify whether the activation of STAT3 is critical for SEA-induced senescence in LX-2 cells, siRNA against STAT3 was transfected into LX-2 cells to suppress STAT3 expression. As illustrated in Fig. 5c, SA-β-gal activity induced by SEA treatment was markedly decreased by knockdown of STAT3. In contrast, knockdown of p53 did not appear to inhibit the expression of STAT3 (Fig. 4b). Taken together, these results indicated that SEA-induced LX-2 senescence via a STAT3-dependent mechanism and that STAT3 was likely to function as an upstream molecule of p53.

### SOCS3 is involved in SEA-induced LX-2 cell senescence through its interaction with p53

**Figure 6:**
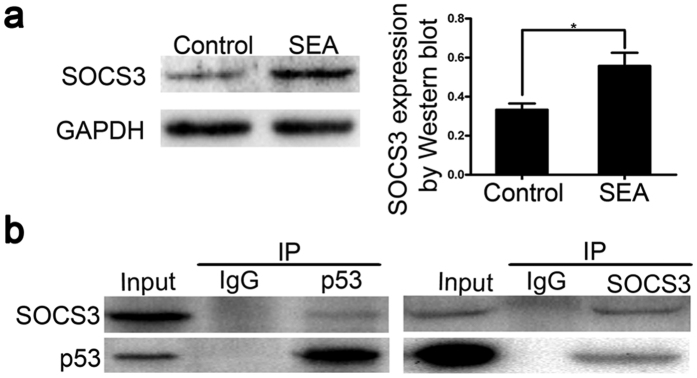
SOCS3 is involved in SEA-induced LX-2 cell senescence through its interaction with p53. (**a**) Western blot analysis for SOCS3 in LX-2 cells treated with or without SEA. (**b**) LX-2 cells were collected and lysed for immunoprecipitation with p53, SOCS3 or IgG control antibodies, followed by Western blotting analysis. *p < 0.05.

Suppressor of cytokine signaling 3 (SOCS3) has been demonstrated to play a special role in the regulation of senescence-associated cell cycle arrest^22^. To investigate whether SOCS3 participates in SEA-induced senescence, we examined the expression of SOCS3 in our system. As shown in Fig. 6a, the protein level of SOCS3 was significantly augmented in SEA-treated group compared to control group. Previous studies have also shown that SOCS3 and p53 directly interact with each other^2^. Therefore, we performed immunoprecipitation to determine if SOCS3 regulated LX-2 senescence triggered by SEA through interacting with p53. Results of the immunoprecipitation assay demonstrated a direct interaction between p53 and SOCS3 (Fig. 6b). These data suggested that SOCS3 was involved in SEA-induced LX-2 cell senescence through interacting with p53.

### SEA promotes senescence of LX-2 cells through activation of TLR4 signaling

**Figure 7:**
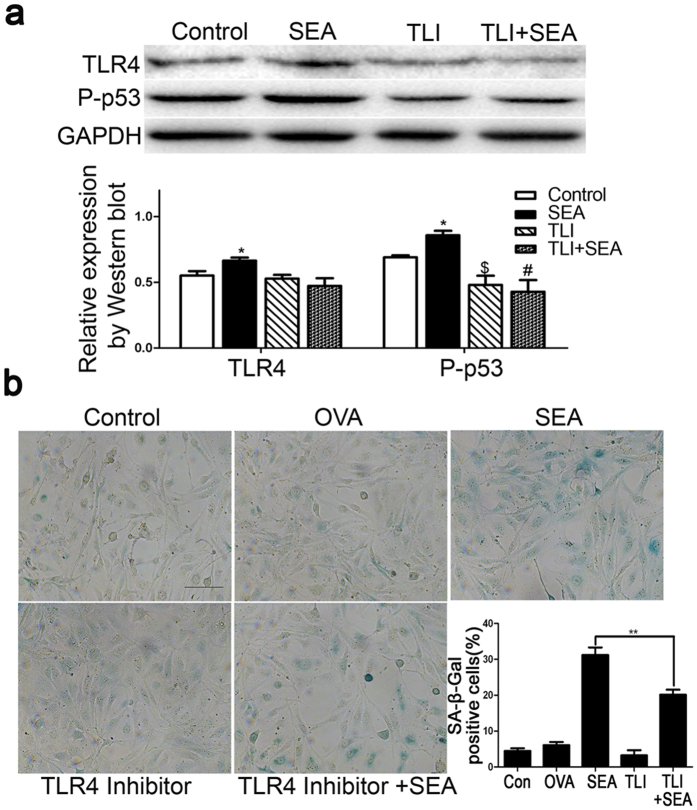
SEA promotes the senescence of LX-2 cells through activation of TLR4 signaling. (**a**) Western blot analysis for P-p53 or TLR4 in LX-2 cells treated with SEA or TLR4 inhibitor TAK-242 (1 nM). *p < 0.05 compared to the control group; ^$^*p* < 0.05 compared to the SEA group; ^#^*p* > 0.05 compared to the TLR4 inhibitor group. (**b**) A representative image from SA-β-Gal staining and percentage (%) of SA-β-Gal -positive cells following SEA or TAK-242 treatment. Bar: 50 micrometers. **p < 0.01.

Recent studies have demonstrated that IL-6 is upregulated in response to oncogene-induced senescence (OIS) and it is required for maintaining senescence^23^. In addition, inhibition of TLR4 decreases the production of IL-6, as well as other senescence-associated secretory phenotypes (SASP)^24^. To identify whether TLR4 is involved in the progression of senescence in LX-2 cells, we examined the expression of TLR4 in LX-2 cells. Western blot analysis suggested that the TLR4 protein levels were upregulated upon SEA stimulation. We then employed TAK-242, a small-molecule-specific inhibitor of TLR4 signaling, which can inhibit the production of lipopolysaccharide-induced inflammatory mediators by binding to the intracellular domain of TLR4, to investigate if altering TLR4 activity affects SEA-induced senescence. As expected, SA-β-Gal activities, as well as the expression of senescence-associated protein P-p53 were inhibited by TAK-242 (Fig. 7). Together, these results indicated that TLR4 might play a role in LX-2 cell senescence triggered by SEA.

## Discussion

Liver fibrosis is a precursor to liver cirrhosis, which is considered as a major health problem worldwide. Liver fibrosis is triggered by chronic liver damage caused by a wide range of etiologies. It was historically considered to be a passive and irreversible process due to the collapse of the hepatic parenchyma and its replacement with a collagen-rich tissue. Nevertheless, accumulating evidence has demonstrated that fibrosis, and even cirrhosis, can be potentially reversed. Activation of resident HSCs to 1) differentiate into myofibroblasts, 2) to proliferate and 3) to produce the network of extracellular matrix remains as the dominant driving force of liver fibrosis. Therefore, approaches that can limit HSCs activation could contribute significantly to the resolution of liver fibrosis^16^,^25^. Studies in rodents have demonstrated that apoptosis in HSCs with an along with an increase in caspase 3 activity or a reduction in Bcl-2/Bax ratio, could promote the regression of CCl_4_ and BDL-mediated liver fibrosis, suggesting a potential role of apoptosis of HSCs in attenuating the disease^26^. In line with these findings, our previous reports have shown that the induction of apoptosis in HSCs is contributive to reverse liver fibrosis induced by *S. japonicum* infection^1^. Therefore, promoting apoptosis of HSCs has emerged as a possible therapeutic strategy for treating liver fibrosis. Apart from the apoptosis of HSCs, recent studies have also shown that senescence of HSCs might also has a similar effect on inhibiting liver fibrosis^8^.

Senescence is a cellular response triggered by various types of stimulation. Once cells become senescent, it undergoes morphologic changes, loses the ability to progress through the cell cycle and gets arrested in the G0/G1 phase. The proliferation capacity of senescent cells reduced remarkably compared to non-senescent cells. Kong X *et al*. has demonstrated that interleukin-22 induces HSCs senescence and restricts liver fibrosis in mice. Genes associated with ECM production are downregulated, yet those related to matrix metalloproteinases are upregulated in senescent HSCs. In addition, HSCs in mice lacking p53 or p16, both key senescence regulators, could continue to proliferate, leading to more extracellular matrix production and ultimately, liver fibrosis^8^. Previous studies have also shown that many genes encoding cytokines (e.g., IL-8) or receptors (MICA, a ligand of NK cell receptor NKG2D) that potentiate natural killer (NK) cell function are upregulated in senescent HSCs. Eventually, the senescent HSCs will be selectively eliminated by NK cells to inhibit liver fibrosis^27^, which indicates senescence of HSCs facilitates their clearance by NK cells. Therefore, senescence of HSCs is of great significance in reversing fibrosis. In our system, SEA might be involved in inhibiting liver fibrosis through triggering senescence in HSCs and decreasing expression of fibrosis-associated proteins such as α-SMA and procollagen I (Figs 1 and 2).

The tumor suppressors, like p53 and p16, play essential roles in multiple responses including growth inhibition, apoptosis and cell cycle arrest^28^. In our previous study, p53 was involved in cell apoptosis in HSCs induced by SEA^1^,^29^. Besides, it works as an essential mediator of senescence in multiple cell types^19^,^30^,^31^. In our study, we focus on the role of SEA on the cellular senescence in HSCs and the associated cell pathway triggered by SEA. Tran D, *et al*. has demonstrated that prolonged insulin-like growth factor (IGF) treatment triggers premature cellular senescence in a p53-dependent manner, and inhibition of p53 prevents IGF-1-induced premature cellular senescence^32^. Besides, fibroblast cell lines overexpressing p16 (INK4A) also show elevated expression of senescence markers, such as cell cycle arrest and senescence-associated β-galactosidase^33^. Cellular senescence in response to DNA damage or dysregulation of mitogenic oncogenes is primarily triggered through the p53 signaling pathway^34^. Additionally, p21^waf1/cip1^, a cell cycle inhibitor, is transcriptionally targeted by p53^35^. In line with these findings supporting the notion that reduction in p53 or p21 prevents the induction of senescence, the LX-2 cells that lack p53 in our study also showed significant decrease in senescence (Fig. 4), suggesting that senescence in LX-2 cells induced by SEA could be mediated by p53. Despite that p16 has been shown to involve in the induction of senescence in a wide variety of cell types, such as gastrokine 1 (GKN1), we did not observe any implication that p16 played a major role in the senescence mediated by SEA^36^.

Signal transducer and activator of transcription 3 (STAT3) has been suggested to be involved in regulating premature cellular senescence. STAT3-related cytokines (e.g., interleukin-6)^37^ activate Janus tyrosine kinases (JAK), and then trigger the translocation of phosphorylated-STAT3 (P-STAT3) to the nuclei, where it functions as transcriptional factor and ultimately induces cellular senescence^38^. Previous studies have reported that knockdown of STAT3 prevents IL-22-induced HSCs senescence, whereas the overexpression of STAT3 promotes HSCs senescence through p53- and p21-dependent pathways *in vitro*^2^. LPS could induce inflammatory mediator production in RAW264.7 cells by STAT1/3 and ERK1/2 signaling pathways^39^. To role out the possibility that LPS triggered activation of STAT3 signaling instead of SEA, the removal of endotoxin from SEA was confirmed as our previously described^29^. In our study, we noticed that P-STAT3 upregulated significantly upon exposure of LX-2 cells to SEA with removal of endotoxin, but not total STAT3. Furthermore, knockdown of STAT3 abolished SA-β-gal activity in SEA-treated LX-2 cells, followed by a significant decline in the levels of P-p53 and p21 (Fig. 5). Taken together, our results demonstrate that expression of P-p53 and p21 is under the control of STAT3, which suggests the possibility that STAT3, together with the p53/p21 axis, constitutes a signaling network that regulates senescence of LX-2 cells.

IL-6/STAT3 signaling has been shown to be essential for the paracrine activity of senescent breast cancer cells^23^. Besides its paracrine mitogenic effect, IL-6 is also required for the execution of OIS in cell-autonomous mode. Knocking down IL-6 causes the inflammatory network to abrogate induction and maintenance of senescence^10^. To date, certain signaling pathways have been suggested to contribute to the induction of IL-6 during OIS, such as transcription factor C/EBPβ, DNA damage responses (DDRs) proteins ATM, NBS1 and CHK2^40^. A few studies have also indicated that the inhibition of TLR4 decreases IL-6 production^24^. Based on these findings, we assumed that TLR4 is involved in SEA-induced senescence. As expected, our results demonstrate that inhibition of TLR4 expression reversed the SEA-induced HSCs senescence observed in this study.

As vital feedback inhibitors of Janus (Jak) kinase signaling, suppressor of cytokine signaling (SOCS) proteins inhibit the expression of inflammatory genes as well as the activation of STAT3 in response to cytokines that belong to the IL-6 family^41^. Previous reports have suggested that the kinetic activation of STAT3 might be the putative cause of the biological effects of SOCS3^42^. SOCS3 is in turn mediated by STAT3, which establishes a negative feedback loop that acts as STAT-induced STAT inhibitors. Apart from these, recent studies have indicated that SOCS3 promoted G1 arrest by acting on the STAT3-p21 pathway^22^. Besides, Kong X, *et al*. has also demonstrated that SOCS3 functions through directly binding to p53, resulting in enhanced expression of p53 and p21. Consistent with this report, we also found that SOCS3 interacted with p53 (Fig. 6), and triggered SEA-induced HSCs senescence, leading to decreased expression of fibrosis-related proteins. Nevertheless, SOCS3 increased the expression of P-p53, but not p53 in our study. Therefore, we conclude that SOCS3 is involved in the induction of SEA-induced senescence.

**Figure 8:**
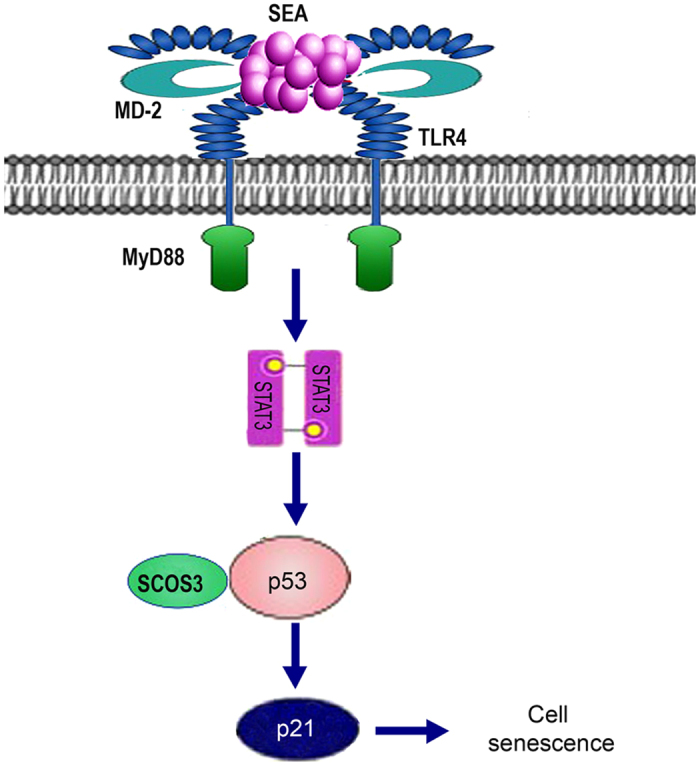
Proposed mechanism of SEA-mediated senescence of LX-2 cells. SEA upregulates the expression of P-STAT3 via the activation of TLR4 signaling. Then, P-STAT3 may contribute to the upregulation of P-p53 as well as its targeting gene, p21. Additionally, SOCS3 is also involved in the process through interaction with p53. Ultimately, the increased level of p21 promotes the senescence of LX-2 cells.

In summary, our findings provide evidence supporting a possible mechanism by which SEA induces senescence in LX-2 cells. In general, SEA might restrict the liver fibrosis by promoting HSCs senescence through the STAT3-p53 pathway, in which SOCS3 is also involved (Fig. 8).

## Methods

### Ethics statement

All animal procedures were performed in accordance with the institutional ethical guidelines for laboratory animal care and use of Nantong University, and conformed to the guide for laboratory animal care and use of Ministry of Science and Technology of China. All animal studies were performed following the protocol number 2014011, which was approved by Animal Care Committee of Nantong University.

### Reagents

SEA of *S. japonicum* and *S. japonicum* cercariae were obtained from Jiangsu Institute of Parasitic Diseases (China). SEA was sterile-filtered and endotoxin was removed with Polymyxin B agarose beads (Sigma, USA). Limulus amebocyte lysate assay kit (Lonza, Switzerland) was used to confirm the removal of endotoxins from the SEA as previously described^29^. Primary antibodies for α-SMA, p53, p21, p16, Toll-like receptor (TLR) 4, SOCS3 and STAT3 were purchased from Santa Cruz Biotechnology (USA). Primary antibody for collagen I was purchased from Abcam. Primary antibodies for phospho-p53 (Ser15) and phospho-STAT3 (Tyr-705) were purchased from Cell Signaling Technology (USA). All of the secondary antibodies were obtained from Santa Cruz Biotechnology (USA). TAK-242, a small-molecule-specific inhibitor of TLR4 signaling, was purchased from Medchemexpress.

### Animals

Male Institute of Cancer Research (ICR) mice were obtained from Center for Experimental Animals of Nantong University (Nantong, China). Each mouse was infected with 20 ± 2 *S. japonicum* cercariae to construct the mouse models infected with *S. japonicum* and sacrificed at 6, 12, 20 weeks respectively. Then the liver tissues were treated with Hematoxylin-eosin staining and SA-β-Gal staining as well as Immunofluorescence analysis. The detailed experimental methods referred to our previous article^43^.

### Cell culture

The human hepatic stellate cell line (LX-2) was purchased from Xiang Ya Central Experiment Laboratory (China). Cells were cultured in Dulbecco’s Modified Eagle Medium (DMEM, Gibco, USA) supplemented with 10% Fetal Bovine Serum (FBS, Thermo, USA) in a humidified incubator at 37 °C with 5% CO_2_.

### RNA interference

After evaluating the knockdown efficiency of four different shRNAs targeted for p53, the following targeting sequences of shRNAs were selected: 5′CCATCCACTACAACTACAT3′ for shRNA-mediated knockdown in LX-2 cells using FuGENE HD (Promega, USA) according to the manufacturer’s instructions. The shRNAs were purchased from Shanghai Genechem Co. Ltd. For STAT3 interference, siRNA duplexes against STAT3 (5′-CCAACGACCUGCAGCAAUA-3′) and control duplex (5′-CCUACGCCACCAAUUUCGU-3′)^44^ were purchased from GenePharma (China). Transfections of the aforementioned siRNAs were carried out using Lipofectamine 2000 reagents (Invitrogen, USA). After 24 h of transfection, the cells were treated with or without SEA for another 48 h and then harvested for Western blot assays.

### Western blot

Equal amounts of cell lysates were separated using 8–12% sodium dodecyl sulfate-polyacrylamide gel electrophoresis (SDS-PAGE) onto 0.45 μM or 0.22 μM polyvinylidene difluoride (PVDF) membranes and blocked with 10% nonfat dry milk at room temperature. The target proteins were incubated overnight with specific primary antibodies at 4 °C. Then, the membranes were washed with TBS containing 0.1% Tween20 and subsequently incubated with horseradish peroxidase (HRP)-conjugated secondary antibodies for 1 h at room temperature. Densitometric analysis of individual bands was performed using Image J software.

### SA-β-Gal staining

The senescence-associated β-galactosidase assay was performed using a Senescence Staining Kit (GenMed Scientifics Inc. USA). Briefly, cells were plated onto 6-well culture plates, treated with SEA for 3 days, then washed once with cleaning solution, and fixed with a fixation solution for 5 min at room temperature. Cells were washed 3 times with an acidic solution and incubated with the staining solution overnight at 37 °C prior to microscopic analysis. The percentage of cells that were stained with senescence-associated β-galactosidase (SA-β-Gal) was determined for each sample using a bright field microscope.

### Immunofluorescence analysis

The cells or tissues were fixed with 4% paraformaldehyde in PBS for 1 h and permeabilized with 0.1% Triton X-100 for 5 min. After blocking in 5% BSA for 2 h at room temperature, the cells were incubated with the primary antibodies against p53 (Santa Cruz Biotechnology, USA), α-SMA (Santa Cruz Biotechnology, USA) or STAT3 (Santa Cruz Biotechnology, USA) and then incubated with the second antibodies. The cells were also stained with Hoechst 33342 (Sigma, USA) for 15 min and examined by confocal laser-scanning or fluorescent microscopy.

### Cell proliferation analysis

LX-2 cells were plated onto 96-well culture plates (5,000 cells per well). After incubation for 24 h, the cells were treated with or without SEA for 96 h. Cell proliferation was measured using 3-(4, 5-dimethylthiazol-2-yl)-2, 5-diphenyltetrazolium bromide (MTT) assay. At the end of the culture process, 10 μl MTT was added to each well. After 4 h of additional incubation, 100 μl DMSO was added to each well and the plate was shaken gently. The absorbance was measured on a Microplate reader at a wavelength of 490 nm.

### Flow cytometry

LX-2 cells were seeded onto 6-well culture plates (2 × 10^5^ cells per well). After incubation in DMEM supplemented with 10% FBS for 24 h, cells were starved in DMEM supplemented with no FBS for another 24 h, followed by treatment with SEA for 48 h. Cell cycle distribution was determined by flow cytometry analysis. For cell cycle analysis, collected cells were washed with cold PBS and fixed in 70% ethanol at −20 °C overnight. After two washed with PBS, the cells were incubated with RNase A at 37 °C for 30 min. Cells were washed with PBS afterward and stained with propidium iodide in dark at room temperature for 30 min. Finally, the cells were subjected to flow cytometry. Cell cycle phases were determined using the Modfit software.

### Immunoprecipitation

For immunoprecipitation, cells were lysed in RIPA buffer for 30 min at 4 °C. An equal amount of each protein (1–2 mg) lysate was incubated with the corresponding antibodies for IP (Santa Cruz Biotechnology) overnight at 4 °C, followed by an incubation with 30 μl of protein A agarose beads (Roche, USA) for 16 h at 4 °C. The immune complexes were analyzed by Western blot analyses with the indicated antibodies.

### Statistical analysis

Data is expressed as mean ± SEM (standard error). A minimum of 6 animals per group was used. All p values were calculated using a two tailed paired Student’s t test or a one way ANOVA. *p* < 0.05 was considered as statistically significant.

## Additional Information

**How to cite this article**: Chen, J. *et al*. Soluble egg antigens of *Schistosoma japonicum* induce senescence in activated hepatic stellate cells by activation of the STAT3/p53/p21 pathway. *Sci. Rep.* **6**, 30957; doi: 10.1038/srep30957 (2016).
